# Analysis of Metabolic Alterations Related to Pathogenic Process of Diabetic Encephalopathy Rats

**DOI:** 10.3389/fncel.2018.00527

**Published:** 2019-01-14

**Authors:** Minjian Dong, Mengqian Ren, Chen Li, Xi Zhang, Changwei Yang, Liangcai Zhao, Hongchang Gao

**Affiliations:** School of Pharmaceutical Sciences, Wenzhou Medical University, Wenzhou, China

**Keywords:** diabetic encephalopathy, nuclear magnetic resonance, metabonomics, lactate, hydroxycarboxylic acid receptor 1

## Abstract

Diabetic encephalopathy (DE) is a diabetic complication characterized by alterations in cognitive function and nervous system structure. The pathogenic transition from hyperglycemia to DE is a long-term process accompanied by multiple metabolic disorders. Exploring time-dependent metabolic changes in hippocampus will facilitate our understanding of the pathogenesis of DE. In the present study, we first performed behavioral and histopathological experiments to confirm the appearance of DE in rats with streptozotocin-induced diabetes. We then utilized nuclear magnetic resonance-based metabonomics to analyze metabolic disorders in the hippocampus at different stages of DE. After 1 week, we observed no cognitive or structural impairments in diabetic rats, although some metabolic changes were observed in local hippocampal extracts. At 5 weeks, while cognitive function was still normal, we then examined initial levels of neuronal apoptosis. The characteristic metabolic changes of this stage included elevated levels of energy metabolites (i.e., ATP, ADP, AMP, and creatine phosphate/creatine). At 9 weeks, significant cognitive decline and histopathological brain damage were observed, in conjunction with reduced levels of some amino acids. Thus, this stage was classified as the DE period. Our findings indicated that the pathogenesis of DE is associated with time-dependent alterations in metabolic features in hippocampal regions, such as glycolysis, osmoregulation, energy metabolism, choline metabolism, branched-chain amino acid metabolism, and the glutamate–glutamine cycle. Furthermore, we observed alterations in levels of lactate and its receptor in hippocampal cells, which may be involved in the pathogenesis of DE.

## Introduction

Diabetes mellitus is a common metabolic disorder that can lead to cognitive decline and dementia. Dejong (1950) introduced the term “diabetic encephalopathy (DE)” to describe cognitive impairment in diabetes. Recent clinical studies have indicated that patients with diabetes exhibit slight structural changes in the cortical gray matter or other domains (Brismar et al., 2007; Jongen and Biessels, 2008). While hyperglycemia is among the factors that contribute to DE (Wessels et al., 2008), several studies have revealed that both vascular and metabolic disturbances (e.g., polyol metabolism, glycation end products) are also potential risk factors (Gabbay et al., 1966; Chung et al., 2003; Mäkimattila et al., 2004; McCrimmon et al., 2012). Diabetes-related cognitive decline is also related to disrupted insulin signaling, oxidative stress, and mitochondrial dysfunction (Ye et al., 2011; Yi et al., 2011). These factors are critical in the development of cognitive impairment in the patients with diabetes. Nevertheless, previous studies have examined these factors at only one time-point during the disease process. The detection of endogenous metabolic variations during the whole diabetic period may aid in elucidating the mechanisms involved in the pathogenesis of DE (Williams et al., 2005).

Metabonomic analysis is one systemic platform used to identify key metabolites related to pathological factors in various disease states. Such methods have been extensively applied to explore potential biomarkers for diseases, providing key insights into their pathogenesis, and to monitor the therapeutic effects of medications (Nicholson et al., 1999; Lu et al., 2013; Chen et al., 2014; Luo et al., 2017). In addition, nuclear magnetic resonance (NMR) analysis exhibits high reproducibility and quantitative merits, with minimal sample preparation (Verpoorte et al., 2008). Using NMR-based metabonomics, we demonstrated that DE is associated with changes in glucose metabolism and disturbances in the glutamate–glutamine cycle in the hippocampus (Zheng Y. et al., 2016). We then applied a ^13^C NMR approach using labeled glucose and acetate, observing alterations in particular metabolic pathways in the brains of diabetic animals (Wang et al., 2015; Zheng et al., 2016b). Recently, we revealed that lactate is among the key factors involved in the pathogenesis of DE in rats (Zhao et al., 2018a). However, the time-dependent metabolic alterations related to the whole pathogenic process of DE remain unclear.

The transition from hyperglycemia to the occurrence of DE is a long-term process accompanied by regulation, compensation, and decompensation periods—each of which exhibits unique metabolic features. Previous studies have indicated that rats with streptozotocin (STZ)-induced diabetes exhibit metabolic characteristics similar to those observed in human beings with diabetes (Zhao et al., 2010). In the present study, we applied NMR-based metabonomics to analyze metabolic alterations in hippocampal extracts from rats with STZ-induced diabetes at 1, 5, and 9 weeks, in comparison with their age-matched controls. The aims of the study were as follows: (*i*) to investigate the metabolic features of different stages of DE and (*ii*) to identify factors associated with the pathogenesis of DE.

## Materials and Methods

### Animals Treatment

Male 7-week-old Sprague–Dawley (SD) rats were purchased from Shanghai SLAC Laboratory Animal, Co., Ltd., and maintained in a temperature- and humidity-regulated SPF colony of the Laboratory Animal Center of Wenzhou Medical University. All animals were housed under a 12-h light–dark cycle with lights on at 08:00. Rats were fed standard rat chow and tap water throughout the study period. All animal experiments were performed in accordance with the National Institutes of Health Guide for the Care and Use of Laboratory Animals and were approved by the Biological Research Ethics Committee/Institutional Animal Care and Use Committee of Wenzhou Medical University (Document No. wydw 2016-0083). All efforts were made to minimize animal suffering and reduce the number of animals used.

After 1 week of habituation, the rats were randomly assigned to the control (*n* = 30) or diabetes group (*n* = 30), and each group was divided into three time-points: 1, 5, and 9 weeks. Diabetic rats were treated with STZ (i.p., 60 mg/kg). First, STZ was freshly prepared in citrate buffer (0.1 M, pH = 4.5) at a single dosage. Animals of the control group were treated with vehicle citrate buffer. Random blood glucose levels were measured from the tail 72 h later using a strip-operated blood glucose meter (One Touch Ultra, Lifescan). Animals with blood glucose levels higher than 16.70 mmol/L were classified as diabetic rats. Blood glucose and body weight were measured weekly between 18:00 and 19:00.

### Spatial Working Memory Test

The Y maze test was conducted to test spatial working memory as previously described (Bak et al., 2017). The Y maze was constructed of black painted wood, with three arms (width: 14 cm, length: 50 cm, height: 25 cm) extending from one center at 120°. There were no visual cues inside the maze, but different extra-maze cues were visible from all three arms to enable spatial orientation. Each rat was placed at the end of one arm and allowed to move freely through the maze during a session lasting 6 min, and its behavior was recorded using a camcorder. Behavioral testing began at 018:30 under dim light. Animals were placed on the same arm facing the wall of the arm, and this arm was designated as the start arm. The inside of the Y maze was cleaned with 70% ethanol between each trial and allowed to dry. Arm entry was defined as the entry of four paws into the arm. The sequence of arm entries was recorded. The rate of spontaneous alternation was calculated by dividing the number of alternations by the number of possible alternations, multiplied by 100, as follows:

$$\begin{array}{c} \mathrm{Alternation}\;(\%)\;=\;[(number of alterations)/(total arm entries-2)]\times 100. \end{array}$$

### Spatial Learning Memory Test

Morris water maze (MWM) was carried out to test learning memory, referred to previous study (Wang et al., 2014). The maze consisted of a black pool (diameter: 200 cm, depth: 50 cm) filled with water (26 ± 2°C). The behavior of rats in the pool was traced using a camera connected to an XR-XM101 analysis system (Xinruan Information Technology, Co., Ltd., Shanghai, China). The pool was divided into four equal quadrants, with four orientations designated as starting positions. The pool contained an escape platform submerged in water (2 cm below the surface), which was camouflaged by mixing non-toxic ink into the water. The platform was placed in a constant location in the middle of southwest or third quadrant. The rats were trained with the hidden platform for 4 days. Each day involved training the rats in the four quadrants at 20 min intervals. Each trial was initiated by placing a rat with its back facing toward the platform at one of the starting points. The trial was terminated when the rat stood on the platform for at least 10 s. However, when the rat did not find the platform within 60 s, it was guided to the platform, where it remained for 20 s. During the spatial navigation test (days 1 to 4), all groups accepted four trainings each day. The swimming distance and latency of the rats to find the platform were measured. Probe trials were performed on the fifth day. Each rat underwent a single probe trial wherein it was allowed to swim for 60 s, with the platform removed from its position. The number of crossings at the original platform location was recorded, along with the swimming distance. Rats were habituated to the testing room for 30 min prior to behavioral analyses. On each day after training, all rats were removed from the pool, dried, and returned to their previous cages.

### Tissue Preparation for Immunofluorescence

To prepare fresh frozen sections for immunofluorescence, animals were anesthetized with 300 mg/kg bodyweight chloral hydrate. Anesthetized animals were perfused with normal saline and 4% paraformaldehyde (PFA) to remove the blood and fix the tissue, following which whole brains were rapidly removed, dehydrated with 20 and 30% sucrose solution, frozen, and stored at -80°C. Frozen tissues were processed in a cryostat (Leica CM1950, Germany), and 6-μm thick sections were cut at -20°C, following which they were thawed onto slides (PCB series, Citoglas, China) and stored at -20°C until use.

Frozen sections were fixed in cold acetone for 10 min, washed in ice-cold PBS, and blocked in 5% BSA for 1 h. Slices were incubated overnight at 4°C with the following primary antibodies: glial fibrillary acidic protein (GFAP, 1:200, Santa), microtubule-associated protein 2 (MAP-2, 1:500, Abcam), and hydroxycarboxylic acid 1 (HCA1, 1:500, Abcam). Samples were washed three times with PBS and incubated in secondary antibodies Alexa Fluor 488 (FITC, green) and Alexa Fluor 546 (TRITC, red, Thermo Fisher Scientific) for 2 h at room temperature. Sections were washed with PBS and samples were stained with DAPI (blue, C1005, Biotime) for 5 min for nuclei visualization. The digital images were collected using an epifluorescence microscope (Eclipse Ts2-FL, Nikon, Japan) equipped with: a S Plan Fluor ELWD 20× Ph1 ADM objective (NA = 0.45, Nikon); total pixel 1920 × 1460; pixel size 4.54 μm × 4.54 μm; using an appropriate filter for TRITC (excitation = 525/50 nm; emission = 597/58 nm; exposure time 1.21 s), FITC (excitation = 470/40 nm; emission = 534/55 nm; exposure time 190 ms), and DAPI (excitation = 365/38 nm; emission = 475/90 nm; exposure time 130 ms); a CoolSnap DYNO camera (Photometrics). Digital images were captured using Nis-Elements D software (Nikon, Japan) and processed by Image J software (1.48 v, NIH). For each case, quantitative data were counted randomly from nine fields of each sample slice. The threshold of fluorescence intensity automatically defined by Image J software, which can calibrate all sections’ optical density in the same standard.

### Apoptosis Assay

Cell apoptosis was examined via terminal deoxynucleotidyl transferase-mediated dUTP-biotin nick-end labeling assay (TUNEL assay) using a cell death detection kit (Roche Diagnostics Corporation, Germany), in accordance with the manufacturer’s protocol. From each hippocampal sample, images of 9 fields were captured randomly. The results were expressed as a percentage of TUNEL-positive cells to total DAPI-stained cells.

### Sample Preparation for NMR Analysis

After fasting overnight, the rats were anesthetized with 10% chloral hydrate and decapitated, following which whole brains were removed. The hippocampus was dissected, rapidly frozen in liquid nitrogen, and stored at -80°C until use. The procedure was performed in accordance with previously described methods (Wang et al., 2015). Prior to analysis, intact frozen tissue was weighed and transferred to a centrifuge tube. Each frozen rat hippocampus was added to a solution containing methanol (4 ml/g) and water (0.85 ml/g). The sample was ground using a tissue homogenizer (FLUKO Equipment, Shanghai). The sample was then vortexed, following which 2 ml/g chloroform was added, and the sample was again vortexed. Then, 2 ml/g chloroform and 2 ml/g water were added to the sample prior to vortexing again. The blend was left on ice or in the fridge for 15 min and then centrifuged at 1,000 *g* for 15 min at 4°C. The supernatant was collected and freeze-dried for 24 h.

### Acquisition of ^1^H NMR Spectra

Nuclear magnetic resonance spectra acquisition parameters referred to our previous study (Zheng et al., 2016a). The dried extract samples were dissolved in 600 μl of D_2_O containing TSP (0.2 mM, sodium salt of 3-trimethylsilylpropionic acid) as a chemical shift reference (δ = 0 ppm) for NMR spectroscopy. The supernatants (550 μl) were transferred into 5 mm NMR tubes after agitation and centrifugation (12,000 rpm, 5 min). The ^1^H NMR spectra were recorded at 298 K on a Bruker AVANCE III 600 MHz NMR spectrometer with a 5 mm TXI probe (Bruker BioSpin, Germany). The ^1^H NMR spectra were acquired via a standard single pulse experiment with water signal pre-saturation, “zgpr” with a spectral width of 20 ppm. The main acquisition parameters included data points, 64 K; relaxation delay, 2 s; spectral width, 12,000 Hz; and acquisition time, 2.65 s per scan. The number of scans was 256. Prior to Fourier transformation, the free induction decays (FIDs) were zero-filled to 64 K and an exponential window function with a line broadening factor of 0.3 Hz was applied. Metabolites were assigned using Chenomx Profiler, a module of Chenomx NMR Suite version 7.7 evolution edition (Chenomx, Inc., Edmonton, AB, Canada), and the Human Metabolome Database (HMDB, version 3.6).

### Data Analysis

All spectra were manually corrected for phase and baseline and referenced to the resonance of TSP at 0 ppm using Topspin (v2.1 p14, Bruker Biospin, Germany). The “icoshift” procedure was performed to align NMR spectra using MATLAB (R2013b, The MathWorks, Inc., Natick, MA, United States) (Savorani et al., 2010). The matrix of chemical shift amplitudes across all samples was calculated using a bucket analysis within MATLAB software. The ^1^H NMR spectra (δ 0.5–10) were divided into regions with an equal width of 0.02 and 0.002 ppm and the following spectral intervals were excluded: 4.60–5.50 ppm (water signal) and 3.35–3.36 ppm (methanol signal). The bucket width of 0.02 ppm was used for multivariate pattern recognition analysis. Another bucket width of 0.002 ppm was also applied to the quantitative analysis.

Multivariate statistical analyses, including unsupervised principal component analysis (PCA) and supervised orthogonal partial least squares projections to latent structures-discriminant analysis (OPLS-DA), were carried out respectively, on the NMR data sets using SIMCA-P^+^ software (version 12.0, Umetrics, Umeå, Sweden). The PCA technique was used to reduce the multi-dimensional data matrices into a two- or three-dimensional component and retain the profound information. The PCA scores plot was used to examine possible groupings, and to detect strong outliers among observations. In the present study, the plots were used to investigate changes in metabolic patterns among control and diabetic animals 1, 5, and 9 weeks after STZ injection. The more complex OPLS-DA model was used to identify the specific discriminant information between classes at each time point. OPLS modeling using male control and littermate diabetic rats at three different time points resulted in three different models. To prevent model overfitting, default seven-round cross-validation was applied with 1/7th of the samples being excluded in each round (Qiu et al., 2010). In addition, in the cross-validated OPLS-DA scores plot, each sample was depicted by two symbols: one for the cross-validated score value (t_cv_) and one for the model score value (t_p_) (Wiklund et al., 2008). A smaller difference between the two score values suggests the stability of the OPLS-DA model. Those with relatively larger scores were discarded. The quality and reliability of the models were assessed based on the parameters *R*^2^X and *R*^2^Q, whose values should be no more than 0.5. For the PCA model, only the value of *R*^2^X was necessary.

### Metabolites in the OPLS-DA Model

To select significant and reliable candidate metabolites, multiple criteria were applied based on variable importance in the projection (VIP) ranking, S-plots, and jack-knifed based confidence intervals (CIJF_jk_). The general purpose and procedure of this selective strategy have been described elsewhere (Ni et al., 2008; Wiklund et al., 2008). Briefly, variables with VIP > 1.0 are considered statistically significant. The S-plot combines the contribution/covariance [Cov (t, X)] and reliability/correlation [Corr (t, X)], can identify differential metabolites (Wiklund et al., 2008). A correlation coefficient (of ± 0.58) was regarded as a cutoff value to select the variables with greater contribution to the discrimination [Corr (t, X)]. Those variables with CIJF_jk_ across zero were excluded. Using multivariate statistical analyses, we selected variables meeting the criteria (i.e., VIP > 1, | Corr (t, X)| > 0.58, and the span of CIJF_jk_ excluding zero) as the most significant and reliable ones. When comparing the outcome of the OPLS models, the SUS-plot plays a key role in visualizing the shared and unique results.

In addition, independent Student’s *t-*tests were implemented in SPSS 13.0 software to more fully determine whether the concentrations of the differential metabolites obtained from OPLS-DA modeling were statistically significant between classes at an analysis of variance (ANOVA) level.

### Statistical Analysis

Data are presented as the mean ± SEM. Student’s *t*-tests were used to compare findings between the two experimental groups, while ANOVA with Scheffe’s *post hoc* tests were used to compare findings among multiple groups. For the Y maze and MWM tests, overall differences between groups were calculated via two-way ANOVA. *P*-values < 0.05 were considered statistically significant. SPSS 13.0 was used for all statistical analyses. The graphs were generated using Prism 5.0 (GraphPad Software, Inc., United States).

## Results

### Behavioral Performance

**FIGURE 1:**
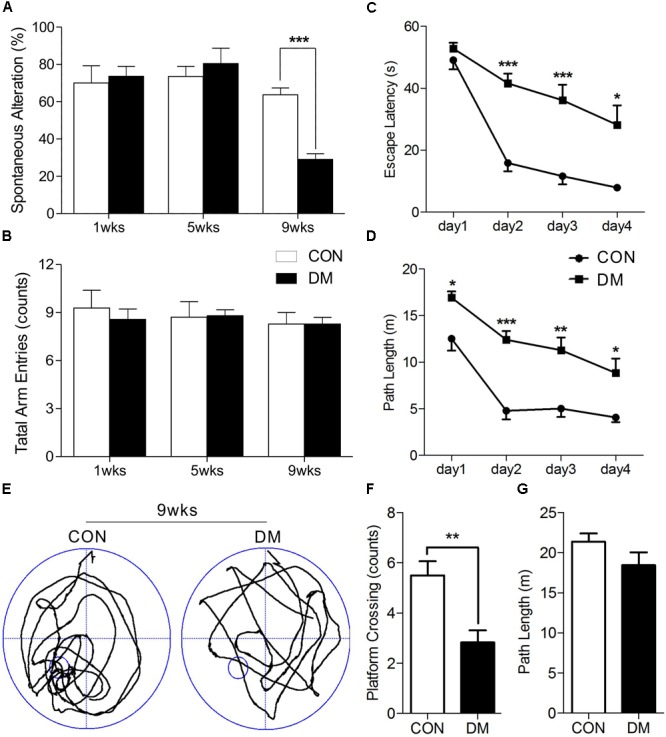
Performance of memory in different stages of diabetic and control rats. Spontaneous alternation behavior **(A)** and the number of arm entries **(B)** in a Y-maze task were measured in diabetic 1-, 5-, and 9-week stages after STZ treatment. Escape latencies **(C)** and path length **(D)** of the diabetic rats in 9-week and age-matched control rats were examined with the Morris water maze (MWM). Representative path tracings **(E)**, number of plateform crossing **(F)**, and path length **(G)** were displayed in probe test. Vertical bars show mean ± SEM. Statistical comparisons were performed with one-way or two-way ANOVA followed by Bonferroni’s *t*-test. *n* = 6–7 for each group. ^∗^*P* < 0.05, ^∗∗^*P* < 0.01, ^∗∗∗^*P* < 0.001, compared to age-matched controls.

After STZ or vehicle injection, rat body weight consistently decreased in the diabetic group, while blood glucose levels significantly increased, suggesting that rats in the STZ group experienced hyperglycemia throughout the experimental period (Supplementary Figure 1). It is well-known that spontaneous alternation in the Y maze reflects working memory, while MWM performance reflects spatial learning and memory. Our results indicated that diabetic rats exhibited learning and memory impairments. A two-way ANOVA (group × time block, repeated measure) revealed a significant group effect on the spontaneous alternation score [*F*_(5,39)_ = 9.342, *P* < 0.001] among the three different time-points, up to a maximum time of 9 weeks after the induction of hyperglycemia. *Post hoc* analysis revealed that working memory had significantly decreased in diabetic rats following 9 weeks of hyperglycemia, when compared to age-matched controls [*t*_(12)_ = 7.193, *P* < 0.001]. However, no such changes were observed at 1 or 5 weeks (Figure 1A). In addition, the number of arm entries remained consistent between the groups at each time point (Figure 1B).

We then used a two-way ANOVA to examine escape latency data for the MWM task. After 9 weeks of hyperglycemia, we observed a significant interaction between group and time block [*F*(1,10) = 6.222, *P* < 0.05], a significant effect of group [*F*(1,10) = 26.539, *P* < 0.001], and time block [*F*(1,10) = 50.054, *P* < 0.001]. Changes in path length produced in training trials in each series of rats exhibited significant main effects of group [*F*(1,10) = 35.068, *P* < 0.001] and time block [*F*(1,10) = 29.019, *P* < 0.001], without significant group-by-trial interactions [*F*(1,10) = 1.210, *P* > 0.05]. *Post hoc* analyses also displayed differences in escape latency and path length between the groups (Figures 1C,D). A 60-s spatial probe trial was examined on day 5, and representative swimming paths are shown in Figure 1E. The number of crossings was significantly lower in diabetic rats than in control rats [*t*(10) = 3.614, *P* < 0.01, Figure 1F], while no significant differences in path length were observed (Figure 1G). Taken together, these data indicate that learning and memory impairments occurred at 9 weeks of hyperglycemia, but not at 1 or 5 weeks.

### Histopathological Examination of Hippocampal Regions

**FIGURE 2:**
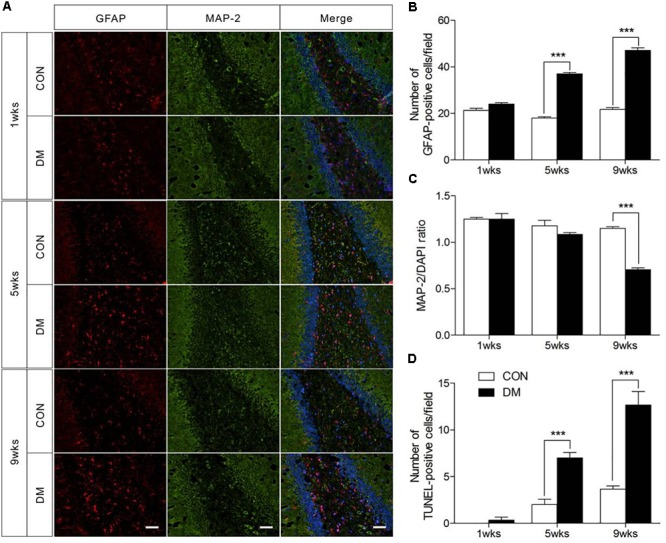
Pathological evaluation of hippocampal cells in different stages of diabetic and control rats. Immunofluorescence staining for GFAP (red), MAP-2 (green), and DAPI staining (blue) in the hippocampal region of diabetic rats **(A)**. Quantification of GFAP staining intensity **(B)**, and MAP-2/DAPI ratio **(C)**, and transferase-mediated dUTP-biotin nick end labeling assay (TUNEL) staining **(D)**. The images of TUNEL were displayed in Supplementary Figure 1. The TUNEL-positive cells in each section was calculated from six random fields. Data were expressed as mean ± SEM, *n* = 3 for each group. Scale bar = 50 μm. ^∗^*P* < 0.05, ^∗∗^*P* < 0.01, ^∗∗∗^*P* < 0.001 compared to age-matched controls.

To determine whether histopathological alterations occur during DE, we examined the immunofluorescence expression of an astroglial marker (GFAP), a neuronal marker (MAP-2), and cellular apoptosis in the hippocampus. As shown in Figure 2, the number of GFAP-positive cells in the hippocampus was significantly increased in diabetic rats at 5 and 9 weeks of hyperglycemia (Figure 2B, *P* < 0.001). In contrast, significantly reduced levels of MAP-2 were observed in the same region in diabetic rats at the 9-week stage only (Figure 2C, *P* < 0.001). Furthermore, TUNEL staining data revealed significant increases in apoptosis in the hippocampus of diabetic rats at 5 and 9 weeks (Figure 2D and Supplementary Figure 2, *P* < 0.001). Overall, these data suggest that astrocyte proliferation and neuronal apoptosis occurred in the hippocampus in diabetic rats at 5 and 9 weeks, but not at 1 week.

### ^1^H NMR Spectra Acquisition and Assignment

**FIGURE 3:**
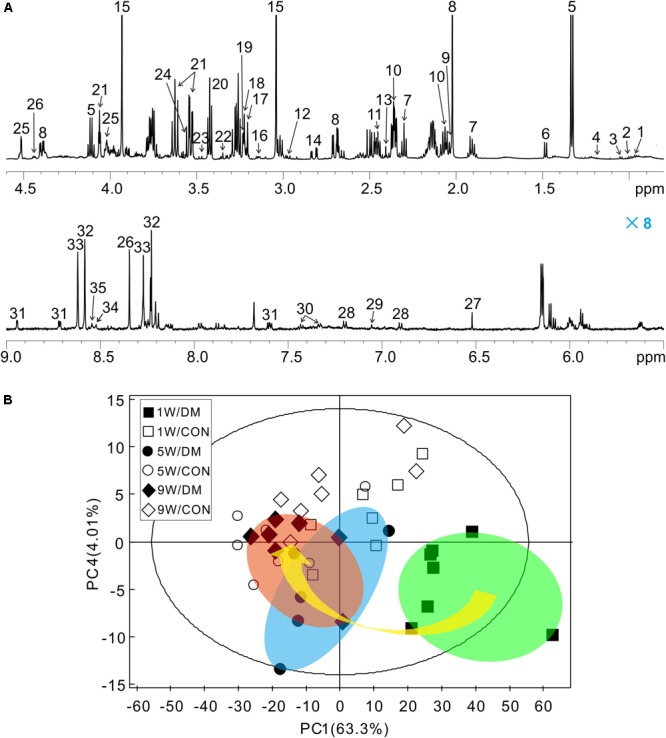
Representative ^1^H NMR spectrum of hippocampus extracts and principal component analysis (PCA). Typical ^1^H NMR spectrum obtained from one rat, spectral regions are 0.8–4.6 and 5.5–9.0 ppm **(A)**. The detail assignments were provided in the Supplementary Table 1. PCA score plot of ^1^H NMR spectra based on all the samples **(B)**. Each symbol in the plot implies one rat. Keys: W, week; DM, diabetes mellitus; CON, control.

Representative ^1^H NMR spectra of hippocampal samples were obtained at different time-points in control (Supplementary Figures 3A–C) and diabetic rats (Supplementary Figures 3D–F). In these spectra, 36 individual metabolites had been assigned and their chemical shift locations are illustrated in Figure 3A (also see Supplementary Table 1). We identified a wide range of amino acids: leucine, isoleucine, valine, alanine, glycine, tyrosine, histidine, phenylalanine; citric acid cycle intermediates: succinate, fumarate; energy substances: lactate, creatine phosphate/creatine (PCr/Cr); carbohydrates: glucose, ethanol, methanol; neurotransmitters: aspartate, glutamate, glutamine, γ-aminobutyric acid (GABA); phospholipids: choline, sn-glycero-3-phosphocholine (GPC), *O*-phosphocholine (PCho), ethanolamine; purines: adenosine, inosine, adenosine 5′-monophosphate (AMP), adenosine 5′-diphosphate (ADP), adenosine 5′-triphosphate (ATP), inosine 5′-monophosphate (IMP); astrocyte-neuron metabolism: *N*-acetyl-aspartate (NAA), *N*-acetyl-aspartyl-glutamate (NAAG); cytoplasmic osmolytes: myo-inositol, taurine, and biological antioxidants: glutathione, ascorbate, niacinamide.

### Multivariate Statistic Analysis

Firstly, a six-component PCA model was obtained from the dataset of the ^1^H NMR spectra. Relative to the more concentrated spectra of controls, the spectra of diabetic rats were more dispersed and exhibited a time-dependent trend from weeks 1 to 9 (arrow, Figure 3B). In the 3D-PCA score plot, metabolic profiles of the control group can be clearly separated from those of diabetic group in the PC1 direction (Supplementary Figure 4). Therefore, these results suggest that the metabolic features of hippocampal tissues may be involved in the progression of DE.

**FIGURE 4:**
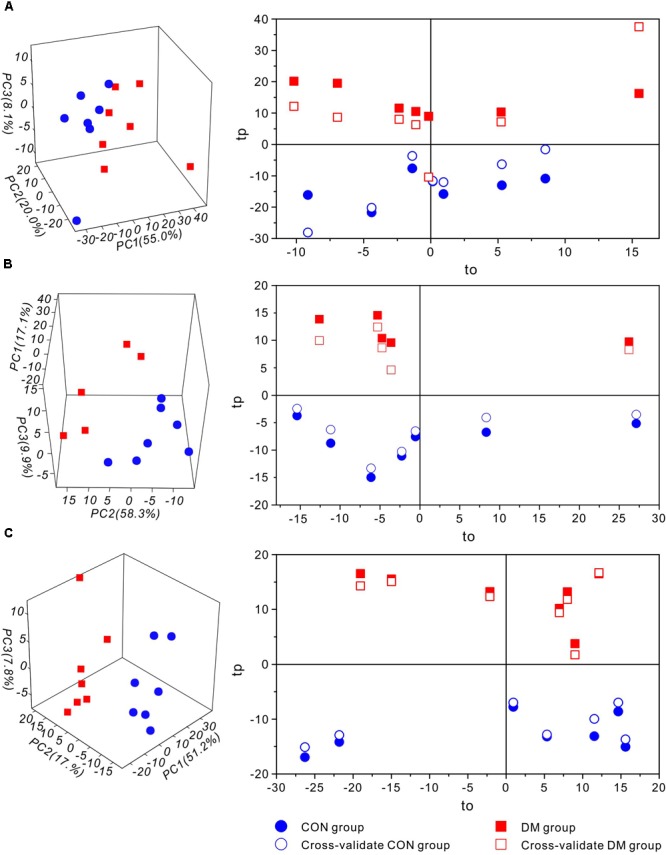
Multivariate statistic analysis of ^1^H-NMR spectra from different stages of diabetic and control rats. 3D-PCA scores plot (left) and 1D cross-validated OPLS-DA score plot (right) of hippocampal extracts from control (CON) and diabetic (DM) rats at diabetic 1-week **(A)**, 5-week **(B)** and 9-week **(C)** stages, respectively. Keys: tp, model score value; to, orthogonal score value; tcv, cross-validated score value.

Then, to further investigate time-dependent metabolic features, 3D-PCA and cross-validated OPLS-DA scores were calculated at three time-points, respectively (Figure 4). Interestingly, in diabetic rats at 1 week, the OPLS-DA score model exhibited overfitting (*P* > 0.05, *R*^2^Q = 0.344), and both the cross-validated score (tcv) and model score (tp) exhibited low overlap, further indicating the instability and unreliability of this OPLS-DA model (Figure 4A). In contrast, after 5 or 9 weeks of hyperglycemia, the 3D-PCA score plot displayed obvious separation between diabetic and control rats, and the cross-validated score for the OPLS-DA models indicated stability and reliability (5 weeks, *P* = 0.0115, *R*^2^Q = 0.809, Figure 4B; 9 weeks, *P* = 0.0003, *R*^2^Q = 0.886, Figure 4C). The model parameters are summarized in Table 1. Taken together, these results suggest that there were significant differences in metabolic features between control and diabetic rats at 5 and 9 weeks, but not at 1 week.

**Table 1:** Summary of the parameters of PCA and OPLS-DA models from different stages of diabetic and control rats.

	PCA models		OPLS-DA models			
Groups	*No*^a^	*R*^2^X(cum)^b^	*No*^a^	*R*^2^X(cum)^b^	*R*^2^Y(cum)^b^	*R*^2^Q(cum)^b^
1 weeks	3	0.832	1P + 1O	0.603	0.914	0.344
5 weeks	3	0.853	1P + 1O	0.740	0.914	0.809
9 weeks	3	0.836	1P + 1O	0.755	0.922	0.886‘
All samples	6	0.904				

*^*a*^Number of components. 1P + 1O, one predictive component and one orthogonal component for constructing the OPLS-DA model. ^*b*^*R*^*2*^X(cum) and *R*^*2*^Y(cum) are the total explained variance in X and Y matrix, respectively, and *R*^*2*^Q(cum) is the cross-validated predictive ability in Y matrix. The values of these parameters close to 1.0 indicate a robust mathematical model with a reliable predictive accuracy.*

### Identification of Characteristic Metabolites

**FIGURE 5:**
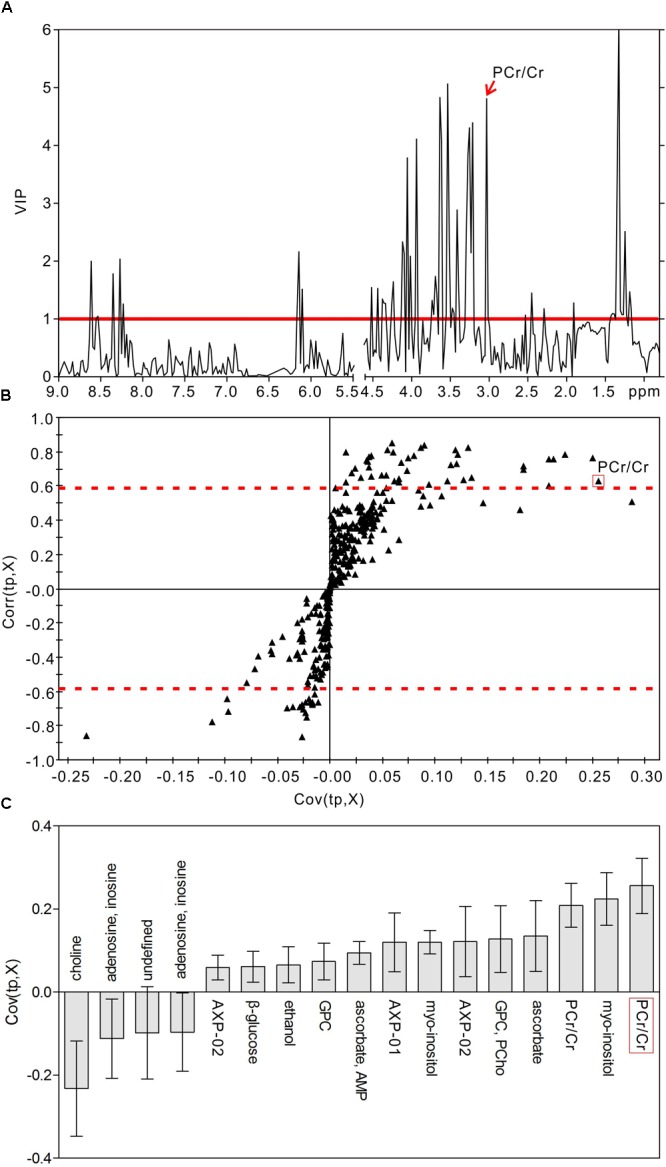
Metabolic features analysis of the hippocampal extracts from the diabetic 5-week rats. VIP plot **(A)**, S-plot **(B)**, and loading plot with CIJF_jk_ **(C)** of OPLS-DA model of ^1^H NMR spectra of diabetic 5-week and control rats. VIP value = 1 was illustrated for red solid line in VIP plot. | Corr (t, X)| = 0.58 was exhibited as red dotted line in S-plot.

**FIGURE 6:**
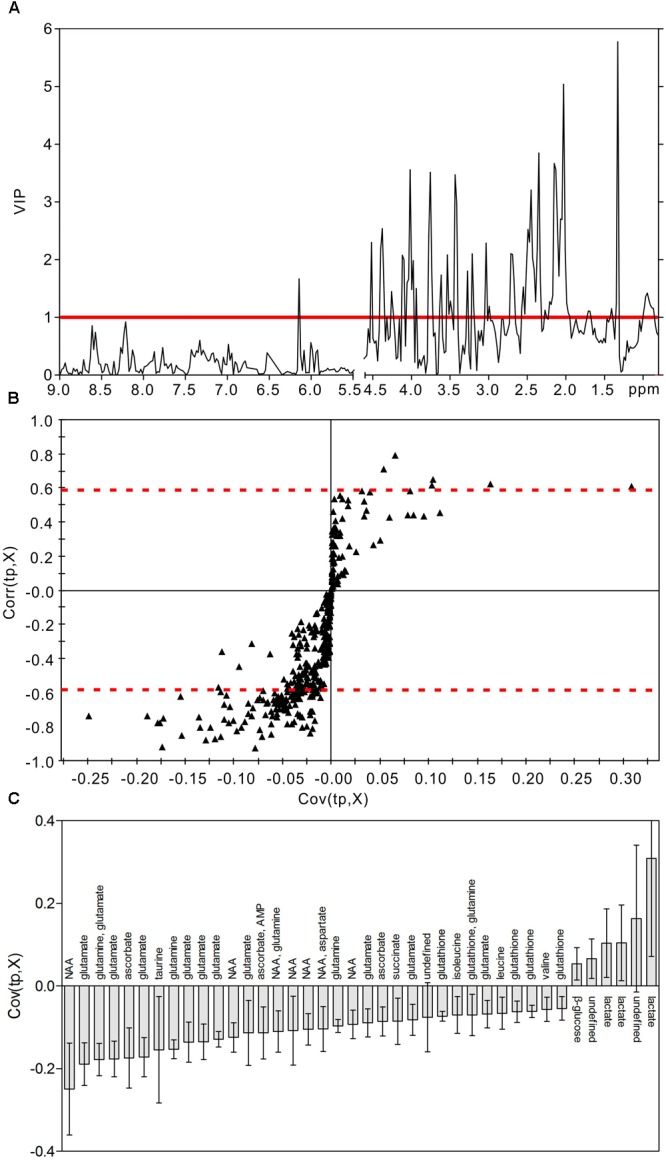
Metabolic features analysis of the hippocampal extracts from the diabetic 9-week rats. VIP plot **(A)**, S-plot **(B)**, and loading plot with CIJF_jk_ **(C)** of OPLS-DA model of ^1^H NMR spectra of diabetic 9-week and control rats. VIP value = 1 was illustrated for red solid line in VIP plot. | Corr (t, X)| = 0.58 was exhibited as red dotted line in S-plot.

In order to identify the metabolic features of the diabetic rat brain, 16 significant variables were screened from the OPLS-DA models. When comparing diabetic and control rats at 5 weeks (Figure 5), the highest VIP value was observed for PCr/Cr, indicating that this variable provided the greatest contribution to class discrimination. In the S-plot, PCr/Cr exhibited a high positive correlation coefficient [Corr (tp, X) = 0.63], indicating that levels were higher in diabetic rats than in controls at 5 weeks. (In contrast, negative correlation coefficients [Corr (tp, X) < 0] indicated that levels were lower in diabetic rats than in age-matched controls.) In the CIJF_jk_ plot, PCr/Cr exhibited a small confidence interval that did not include zero. Positive correlations were observed for the following other metabolites: myo-inositol, ascorbate, GPC, PCho, AMP, ADP, ATP, ethanol, glucose. In addition, negative correlations were observed for adenosine, inosine, and choline. Similarly, in the 9-week OPLS-DA model, 13 metabolites were identified (Figure 6). These included energy metabolites (lactate, glucose), neurotransmitters (glutamate, glutamine, aspartate), amino acids (leucine, isoleucine, valine, taurine, succinate), NAA, AMP, and ascorbate. All correlation coefficients were negative, with the exception of those for lactate and glucose. Taken together, these data illustrate the metabolic features of diabetic rats at 5 and 9 weeks as determined using the OPLS-DA models.

Furthermore, the results of our quantitative analysis (Table 2) were mostly consistent with the results of the OPLS-DA model. We observed that levels of lactate and glucose were increased throughout the hyperglycemic period, while levels of tyrosine were decreased. Interestingly, at 9 weeks, we observed sudden downward shifts in several metabolites that had exhibited increases at 1 or 5 weeks: BCAAs (valine, leucine, isoleucine), glutamate, glutathione, choline, glycine, ascorbate, aspartate, IMP, and energy metabolites (ATP, ADP, AMP, Supplementary Figure 5). Thus, these metabolic changes may reflect the hippocampal response to hyperglycemia, rather than processes directly associated with DE.

**Table 2:** Relative values of metabolites changed in diabetic rats, compared to age-matched controls.

Key	Metabolite	1 week	5 week	9 week
1	Leucine	↑19%	–	↓25%^∗^
2	Isoleucine	↑21%	–	↓26%^∗∗^
3	Valine	↑24%^∗^	↑10%	↓23%^∗∗^
4	Ethanol	–	↑46%^∗^	↓13%
5	Lactate	↑18%^∗^	↑18%	↑29%^∗^
6	Alanine	–	–	–
7	GABA	↑24%	–	–
8	NAA	–	–	↓18%^∗∗^
9	NAAG	–	–	↓18%^∗^
10	Glutamate	↑11%^∗^	–	↓15%^∗∗^
11	Glutamine	–	–	↓23%^∗∗∗^
12	Glutathione	↑10%	–	↓16%^∗∗∗^
13	Succinate	–	↑11%	↓31%^∗∗^
14	Aspartate	↑22%^∗^	–	↓15%^∗^
15	PCr/Cr	–	↑11%^∗^	–
16	Ethanolamine	↑25%^∗∗^	↑13%	–
17	Choline	↑14%	↓53%^∗∗^	↓11%
18	PCho	↑16%	–	–
19	GPC	↑14%	↑20%^∗^	–
20	Taurine	↑18%^∗^	↑17%^∗^	–
21	Myo-inositol	↑31%^∗∗∗^	↑20%^∗^	–
23	β-Glucose	↑30%^∗^	↑37%^∗^	↑30%^∗∗^
24	Glycine	↑15%	↓11%	↓10%
25	Ascorbate	↑10%	–	↓17%^∗^
26	Adenosine, inosine	↑18%	↓29%^∗^	–
27	Fumarate	↑28%^∗^	–	↑35%^∗^
28	Tyrosine	↓39%^∗^	↓43%^∗∗^	↓26%^∗^
29	Histidine	–	↓12%	↓26%^∗^
30	Phenylalanine	–	↓14%	↓29%^∗∗^
31	Niacinamida	↑14%^∗^	–	–
32	IMP	↑38%^∗^	–	↓24%^∗^
33	AMP	–	↑38%^∗^	↓29%^∗^
34	ADP	–	↑36%^∗^	↓14%
35	ATP	–	↑126%^∗∗∗^	↓20%

*Keys: arrows present increased or decreased levels compared to age-matched controls, while dash means the value lower than 10%. ^∗^*P* < 0.05, ^∗∗^*P* < 0.01, ^∗∗∗^*P* < 0.001, compared to age-matched controls, independent samples Student’s *t*-test.*

### Glycolysis and Lactate Receptor

**FIGURE 7:**
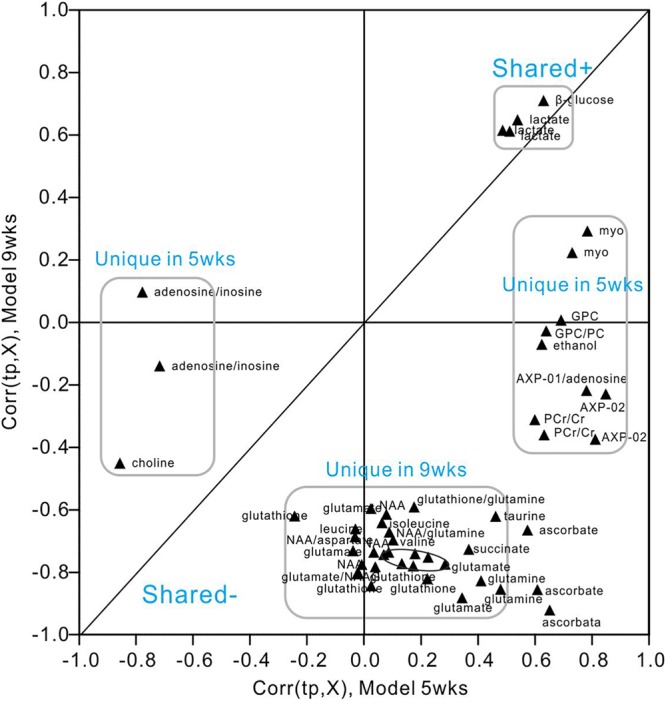
SUS-plot of OPLS-DA models of the diabetic 5- and 9-week rats. The plot was constructed based on both OPLS-DA models, diabetic 5-week versus controls and diabetic 9-week versus controls. Metabolites were found close to either the X or Y axis represented unique for the 5-week model and 9-week model, respectively. Metabolites close to diagonal line represented shared for both models. All the metabolites that were not changed significantly according to the confidence interval in the Figures 5, 6 were removed in this plot to enhance visualization.

We utilized SUS-plots to compare shared and unique metabolites at 5 and 9 weeks of hyperglycemia (Figure 7). In SUS-plots, the correlations from the predictive components [Corr (tp, X)] are plotted. Unique effects are located close to either the X or Y axis, while shared effects are located on the diagonals. In addition, a value of Corr > 0 (+) indicates metabolites that are more abundant in the diabetic group than in the control. Then, metabolites that are abundant in the control group are showed as values of Corr < 0 (-). The following unique metabolites were identified in the 5-week model: choline (-), adenosine (-), inosine (-), creatine (+), myo-inositol (+), AMP (+), GPC (+), ethanol (+), ATP (+), ADP (+). The following unique metabolites were identified in the 9-week model: NAA (-), glutamate (-), glutamine (-), taurine (-), aspartate (-), succinate (-), glutathione (-), leucine (-), isoleucine (-), valine (-). Interestingly, two metabolites (lactate and glucose) were shared by both models, both of which were upregulated in diabetic hippocampal extracts at 5 and 9 weeks. Furthermore, a significant positive correlation was observed between the two, suggestive of increased glycolytic activity (*r* = 0.610, *P* < 0.05, Supplementary Figure 6).

**FIGURE 8:**
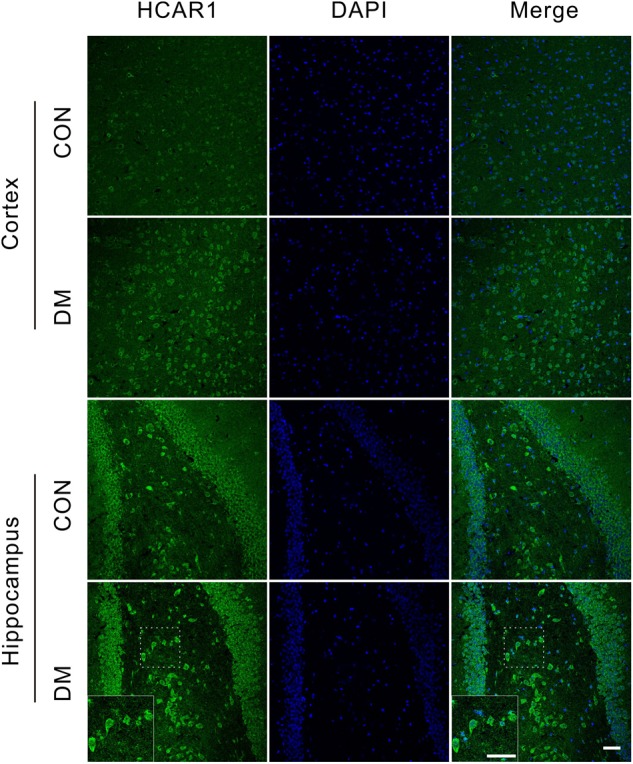
Representative images of HCA1 receptor expressions. Fluorescent staining visualization revealed significantly higher HCA1 expressions (GFP) in the cortex and hippocampus regions of the diabetic 9-week rats compared to age-matched controls. Images are representative of three experiments. Cell nuclei was stained by DAPI (blue). Scale bar = 50 μm.

To investigate changes in the lactate receptor [hydroxycarboxylic acid receptor 1 (HCA1)] in diabetic rats, we performed an immunofluorescence assay detect the receptor expression in the brains of different rat groups (Figure 8). We observed high HCA1 expression in the cortex and hippocampus of control rats, mainly at the cell membrane and within the cytoplasm. However, significant increases in HCA1 expression were observed in diabetic rats at 9 weeks (Supplementary Figure 7), suggesting that the chronic diabetic period is associated with increases in HCA1 levels.

## Discussion

Diabetes is characterized by metabolic disorders that extensively affects multiple organs. Recent studies have highlighted the effect of diabetes on the brain, as the disease has been associated with both cognitive dysfunction and cerebral neurophysiological changes (Brands et al., 2005). In the present study, we investigate the onset of learning and memory impairments in diabetic rats using the Y maze and MWM tests, respectively. Our results indicated that these impairments occurred at the 9-week stage, but not at the 1- or 5-week stages, consistent with the findings of previous studies (Shingo et al., 2012).

In the brain, MAP2 is widely regarded as a neuronal marker, while GFAP is regarded as an astrocyte marker (Eng and Ghirnikar, 1994). In the present study, we observed an increased number of GFAP-positive cells in the hippocampus of diabetic rats at 5 and 9 weeks, suggestive of astrocyte proliferation. These findings are also consistent with previous studies regarding diabetes-related memory impairments in rats (Duarte et al., 2012). Conversely, we observed decreased levels of MAP2, which may be related to increased rates of apoptosis in diabetic rats. Such findings were confirmed via TUNEL assays, which can detect DNA fragmentation (Kyrylkova et al., 2012).

In this study, the ^1^H NMR spectra of hippocampal extracts supplied metabolic information associated with the different stages of diabetes. The observed metabolite variations allowed us to identify key information related to the pathogenesis of DE. Supplementary Figure 8 illustrates the metabolic pathways involved in the pathogenic process of DE based on the KEGG database.

### Metabolic Characteristics of Diabetic Rats at 1 Week

One week after STZ injection, we observed no abnormalities in cognitive ability or histopathology in the hippocampus of diabetic rats, except for changes in levels of metabolites detected via NMR examination. Levels of most of these metabolites, including neurotransmitters and cytoplasmic osmo-regulators, were elevated in the hippocampus, suggestive of an acute response to hyperglycemic stress in this region. For example, both taurine and myo-inositol are important osmolyte modulators (Brand et al., 1993; Bitoun and Tappaz, 2000), and previous studies have indicated that elevated synthesis of osmolytes may be due to increased osmoregulation in the brain following exposure to hyperglycemia (Zheng Y. et al., 2016). Besides, elevated taurine levels have also been associated with anti-oxidative stress (Gao et al., 2012). Furthermore, myo-inositol has been regarded as a marker of astrocytes (Pascente et al., 2016), consistent with the astrocyte proliferation observed via GFAP staining in the present study. Therefore, the 1-week stage may reflect the acute stress in early diabetes.

### Metabolic Characteristics of Diabetic Rats at 5-Weeks

Five weeks after STZ treatment, although no obvious cognitive deficits were observed in diabetic rats, they exhibited significant astrocyte proliferation and neuronal apoptosis in the hippocampus, suggestive of early pathological changes. At the time-point, we observed significant increases in levels of energy substances (i.e., ATP, ADP, AMP, PCr/Cr), but not at 1 or 9 weeks, indicating that this stage included the most active period of energy metabolism. On the one hand, high-energy phosphate compounds (ATP, ADP, AMP) are crucial for sustaining brain function. On the other, the PCr/Cr system, which is linked to adenine nucleotides in the creatine phosphokinase reaction, is also energy reservoir. It is well-known that the human brain consumes 20% of the total energy in the resting state (Belanger et al., 2011). Maintenance and restoration of ion gradients, and uptake of neurotransmitters, are the main contributors to the high energy demands of the brain (Alle et al., 2009). In this stage, activation of energy substances may impede the induction of DE in the diabetic state.

In addition, the metabolic characteristics of this stage also included a decrease in choline content, accompanied by an increase in GPC. Choline plays a key role in phospholipid synthesis in cell membranes (Michel et al., 2006), and their metabolic changes in the hippocampus may be related to the proliferation of astrocytes or neuronal apoptosis. Previous studies have also reported that phospholipid abnormalities may underlie the pathogenesis of Alzheimer’s disease (Pettegrew et al., 2001), and that choline deficiency adversely impacts cognitive ability (Sanders and Zeisel, 2007). Hyperglycemia may thus stimulate these metabolic features in an effort to protect the hippocampus from adverse factors, allowing cognitive function to remain normal.

### Metabolic Characteristics of Diabetic Rats at 9-Weeks

At the 9-week stage, we observed significant impairments in memory and increases in neuronal apoptosis in diabetic rats. The metabolic changes observed in the hippocampus may be directly related to DE. Interestingly, we observed a downward shift in levels of most metabolites, in contrast to the previous two stages. We first observed decreases in hippocampal levels of NAA and NAAG, which are metabolic markers reflecting the functional status of neurons. In addition, levels of energy metabolites such as ATP and PCr were no longer elevated, suggesting depletion of energy metabolites during the DE period, which may have contributed to cognitive decline.

In addition to alterations in energy metabolism, we observed decreases in amino acids levels in hippocampal extracts from diabetic rats at 9 weeks. Such decreases were accompanied by decreases in body weight, indicating that the diabetic condition was associated with attenuation of protein synthesis and altered amino acid metabolism (Teahan et al., 2011). Markedly decreased levels of branched-chain amino acids (BCAAs, including valine, leucine, and isoleucine) were consistent with our previous findings in diabetic rats (Diao et al., 2014). We also observed reduced levels of glutamate and glutamine, in accordance with findings reported for *db/db* mice with DE (Zheng Y. et al., 2016), and mouse models of depression (Veeraiah et al., 2014). The glutamate-glutamine cycle is crucial for brain function, as glutamate is an important neurotransmitter that is released from the pre-synaptic nerve terminal and interacts with corresponding receptors (e.g., *N*-methyl-D-aspartate receptors) (Lyoo et al., 2009). Tyrosine and phenylalanine, which involved in the tyrosine metabolism pathway, are precursors of some biologically active substances, such as catecholamines and thyroid hormones (Levine and Conn, 1967). However, the causal relationship between tyrosine metabolism and DE remains unclear. Taken together, these findings indicate that the 9-week stage is characterized by the exhaustion of energy substances and amino acids, which is called DE period.

### Glycolysis and DE

Our findings indicated that the pathogenic progression of DE is associated with increases in lactate and glucose levels in the hippocampus, which can be observed throughout the disease course. In addition, we observed a significant positive correlation between them, suggestive of enhanced glycolytic activity in the diabetic state. This finding is consistent with those of a previous study involving type 2 diabetic mice with memory impairments (Zheng Y. et al., 2016). Recent studies have revealed that lactate is not only a source of energy, but also acts as a signaling molecule and modulates neuronal functions including excitability, plasticity, and memory consolidation (Mosienko et al., 2015; Magistretti and Allaman, 2018). Suzuki et al. (2011) confirmed that glycogenolysis (the breakdown of glycogen) and the release of its downstream product lactate from astrocytes are required for memory consolidation in response to a learning paradigm. However, the causal relationship between lactate and memory remains unclear, and the role of lactate in the pathogenesis of DE remains to be clarified.

**FIGURE 9:**
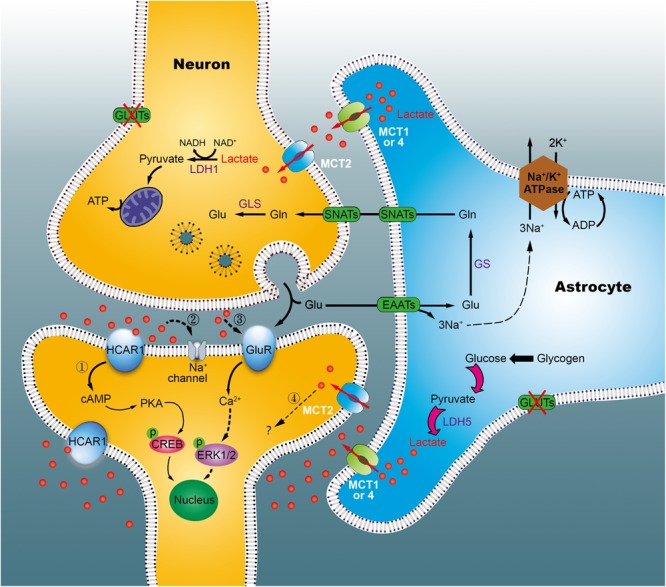
Working model of the astrocyte-neuron lactate shuttle (ANLS) in chronic diabetic conditions. Glucose is taken up by astrocytes and neurons from surrounding capillaries via glucose transporters (GLUT). In cytoplasm, pyruvate can be transported into the mitochondria for TCA cycle and energy synthesis, or converted into lactate, and exported out of the astrocyte by the monocarboxylate transporter (MCT). In neurons, lactate is converted back into pyruvate to generate ATP in mitochondria, which support memory formation and normal function. However, in the chronic diabetic term, we identified that lactate was concomitantly elevated significantly. Excessive lactate accumulated, which may either link to the hydroxycarboxylic acid receptor 1 (HCA1, marked 1) or other mechanisms (marked 2–4). Glu, glutamate; GLS, glutaminases; Gln, glutamine; SNATs, sodium-coupled neutral amino acid transporter; EAATs, excitatory amino acid transporters; LDH, lactate dehydrogenase; GluR, glutamatergic receptors.

By the astrocyte-neuron lactate shuttle (ANLS) hypothesis, it was known that in astrocytes, glucose is predominantly metabolized to lactate, then transported to the extracellular compartment via monocarboxylate transporters 1 or 4 (MCT1, 4) in physiological states (Pellerin and Magistretti, 1994). However, the hypothesis has long been controversial, due to distinct cellular expression of lactate dehydrogenase (LDH), and different kinetic parameters of MCTs on both neurons and astrocytes (Díaz-García et al., 2017; Bak and Walls, 2018). Our studies has indicated that excess lactate is produced in a time-dependent fashion in the hippocampus of diabetic rats (Zheng Y. et al., 2016). Furthermore, using *in vitro* cultures of primary cells, we demonstrated that astrocytes, but not neurons, excrete more lactate under hyperglycemic conditions (Wang et al., 2018; Zhao et al., 2018b). Simultaneously, using [2-^13^C]acetate and [1-^13^C]glucose as tracer substrates, we observed enhancement of the pyruvate recycling pathway in diabetic rats at 1 week, but not at 15 weeks, suggestive of reduced utilization of lactate in the chronic diabetic stage (Wang et al., 2015). Nevertheless, it remains unclear if astrocytes outpace neurons in lactate production, especially during diabetic state. Based on our data, we speculated that, in the early stages of diabetes, astrocytes (including neurons) synthesize more lactate to supply neurons with energy via the pyruvate recycling pathway, thus maintaining normal cognitive function (Figure 9). However, in the late stage, excess lactate may not be fully utilized by neurons, resulting in lactate accumulation outside of cells. Such accumulation may produce adverse effects on cognitive ability via multiple mechanisms, such as the membrane HCA1 receptor-coupled pathway (Bozzo et al., 2013), ionic-related sodium currents (Herrera-Lopez and Galvan, 2018), a cellular redox state (Magistretti and Allaman, 2018), NMDA receptors (Yang et al., 2014), or nitric oxide pathways (Coco et al., 2013). Recently, we revealed that HCA1-PKA-CREB-dependent signaling plays a key role in the pathogenesis of diabetes (Zhao et al., 2018a). However, increased HCA1 expression in the hippocampus of diabetic rats at 9 weeks may be related to positive feedback regulation, suggesting the involvement of other mechanisms in this process. Currently, we are establishing HCA1 knockout mice to explore the precise mechanisms underlying these effects.

## Conclusion

Analyzing metabolic features at different stages of diabetes, we identified three distinct disease periods. Each period was associated with characteristic metabolic alterations in the hippocampus, with the exception of lactate and glucose. In future studies, we aim to determine the causal link between lactate and DE using molecular biology experiments and gene knockout animals.

## Author Contributions

LZ contributed to experimental design and writing of the manuscript. MD contributed to animal experiment data acquisition. HG, CL, XZ, CY, and MR contributed to data analysis and result interpretation. All authors have read and approved the final manuscript.

## Conflict of Interest Statement

The authors declare that the research was conducted in the absence of any commercial or financial relationships that could be construed as a potential conflict of interest.
