# Supplementary material for: Analysis of Metabolic Alterations Related to Pathogenic Process of Diabetic Encephalopathy Rats
Source: Front Cell Neurosci. 2019 Jan 14;12:527. doi: 10.3389/fncel.2018.00527 (PMC6339875; doi:10.3389/fncel.2018.00527)
Supplement: Supplementary file 1 [file Data_Sheet_1.doc]

**Supplementary material**

**Supporting Table 1 Metabolites assignments in 600 MHz ^1^H NMR spectra.**

| Key | Metabolites | Group | δ^1^H (multiplicity)^e^ |
| --- | --- | --- | --- |
| 1 | leucine | δCH_3_; δ'CH_3_ | 0.96(d); 0.97(d) |
| 2 | isoleucine | γCH_3_; δCH | 0.94(t); 1.01(d) |
| 3 | valine | γCH_3_; γ'CH_3_ | 0.99(d); 1.05(d) |
| 4 | ethanol | CH_3_ | 1.19(t) |
| 5 | lactate | βCH_3_; αCH | 1.33(d); 4.11(q) |
| 6 | alanine | βCH_3_ | 1.48(d) |
| 7 | GABA | γCH_2_; βCH_2_; δCH_2_ | 1.90(qu); 2.30(t); 3.02(m) |
| 8 | N-acetyl-aspartate (NAA) | CH_3_; γCH_2_; βCH_2_ | 2.02(s); 2.50(dd), 2.70(dd); 4.39(dd) |
| 9 | N-acetyl-aspartyl-  glutamate (NAAG) | CH_3_ | 2.03(s) |
| 10 | glutamate | γCH; δCH_2_; βCH_2_ | 2.06(m), 2.13(m); 2.35(m), 2.36(m); 3.76(dd) |
| 11 | glutamine | γCH; δCH_2_; βCH_2_ | 2.13(m), 2.16(m); 2.45(m), 2.47(m); 3.77(t) |
| 12 | glutathione | γCH_2_; δCH_2_; 7'CH_2_; 7CH | 2.17(m), 2.18(m); 2.55(m), 2.57(m); 2.93(dd), 2.98(dd); 4.58(dd) |
| 13 | succinate | βCH_2_; γCH_2_ | 2.41(s); 2.41(s) |
| 14 | aspartate | γCH_2_; βCH_2_ | 2.67(dd), 2.82(dd); 3.90(dd) |
| 15 | creatine phosphate/  creatine (PCr/Cr) | N(CH3); βCH_2_ | 3.04(s); 3.93(s) |
| 16 | ethanolamine | βCH_2_ | 3.14(m) |
| 17 | choline | N(CH_3_)_3_ | 3.21(s) |
| 18 | O-phosphocholine | N(CH_3_)_3_; αCH_2_ | 3.22(s); 4.17(m) |
| 19 | sn-glycero-3-phospho  choline (GPC) | N(CH_3_)_3_; 7CH_2_ | 3.23(s); 4.33(m) |
| 20 | taurine | βCH_2_; αCH_2_ | 3.26(t); 3.42(t) |
| 21 | myo-inositol | 5CH; αCH, γCH; δCH, 6CH; βCH | 3.28(t); 3.53(dd); 3.63(t); 4.06(t) |
| 22 | methanol | CH_3_ | 3.36(s) |
| 23 | β-glucose | 5CH | 3.47(m) |
| 24 | glycine | βCH_2_ | 3.56(s) |
| 25 | ascorbate | α'CH_2_; β'CH; δCH | 3.74(m), 3.74(m); 4.02(m); 4.51(d) |
| 26 | adenosine, inosine | γ'CH^a^; α'CH^a^; 8CH_2_^b^ | 4.44(dd); 8.34(s) |
| 27 | fumarate | βCH, β'CH | 6.52(s) |
| 28 | tyrosine | 3,5CH^c^; 2,6CH^c^ | 6.90(m); 7.20(m) |
| 29 | histidine | 5CH^c^ | 7.06(s) |
| 30 | phenylalanine | 2,6CH^d^; 4CH^d^; 3,5CH^d^ | 7.33(m); 7.38(m); 7.43(m) |
| 31 | niacinamide | 5CH; 4CH; 6CH; 1CH | 7.60(dd); 8.25(dd); 8.94(dd) |
| 32 | IMP | 2CH^b^; 8CH^b^ | 8.23(s); 8.56(s) |
| 33 | AMP | 2CH^b^; 8CH^b^ | 8.27(s); 8.60(s) |
| 34 | ADP | 8CH^b^ | 8.52(s) |
| 35 | ATP | 8CH^b^ | 8.54(s) |

^a^Chemical shifts for the group of adenosine from furan ring;

^b^Chemical shifts for the group of adenosine, IMP and AMP from purine ring;

^c^Chemical shifts for the group of tyrosine from Phenyl ring;

^d^Chemical shifts for the group of histidine from Imidazole ring;

^e^s, singlet; d, doubles; t, triples; m, multiplets; qu, quartet; dd, double doublet.


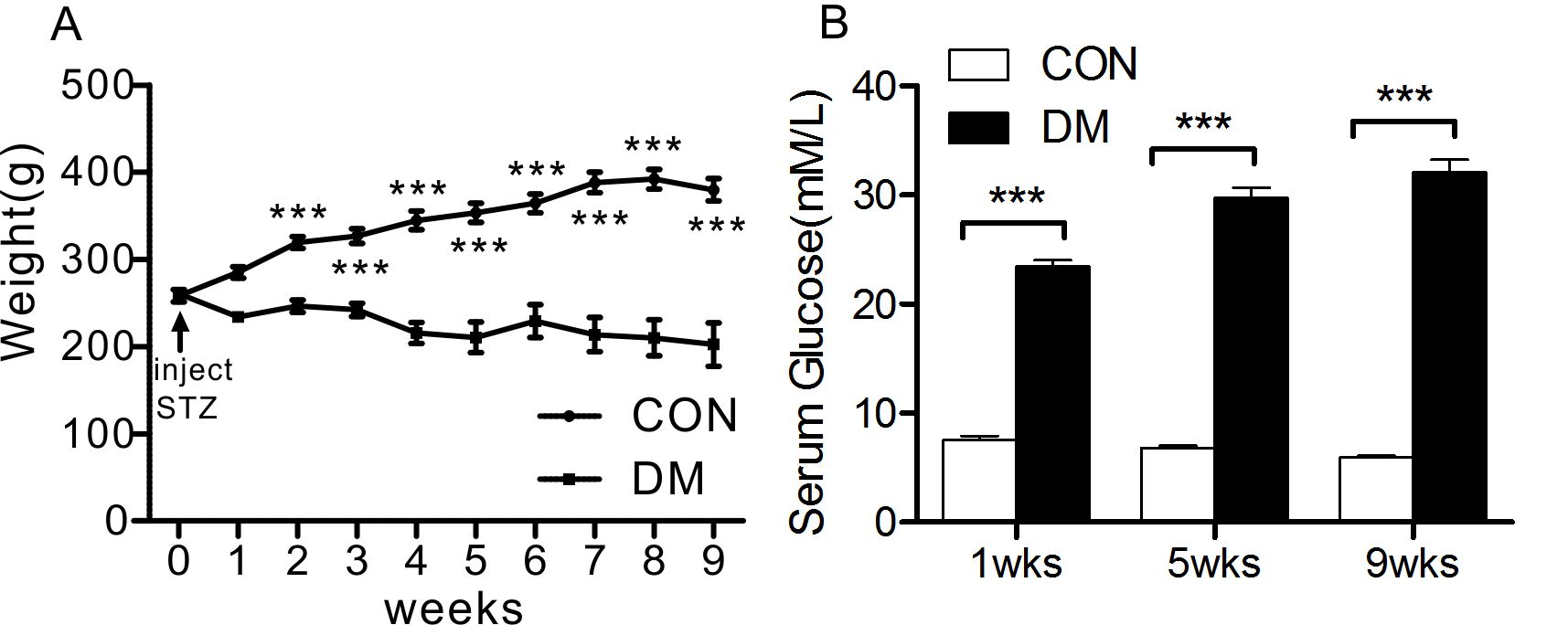


**Supporting Figure 1.** Body weight (A) and serum glucose levels (B) of the rats from the diabetic 1- to 9-week and controls. Data are expressed as mean ± SEM. ^***^*p* < 0.001.


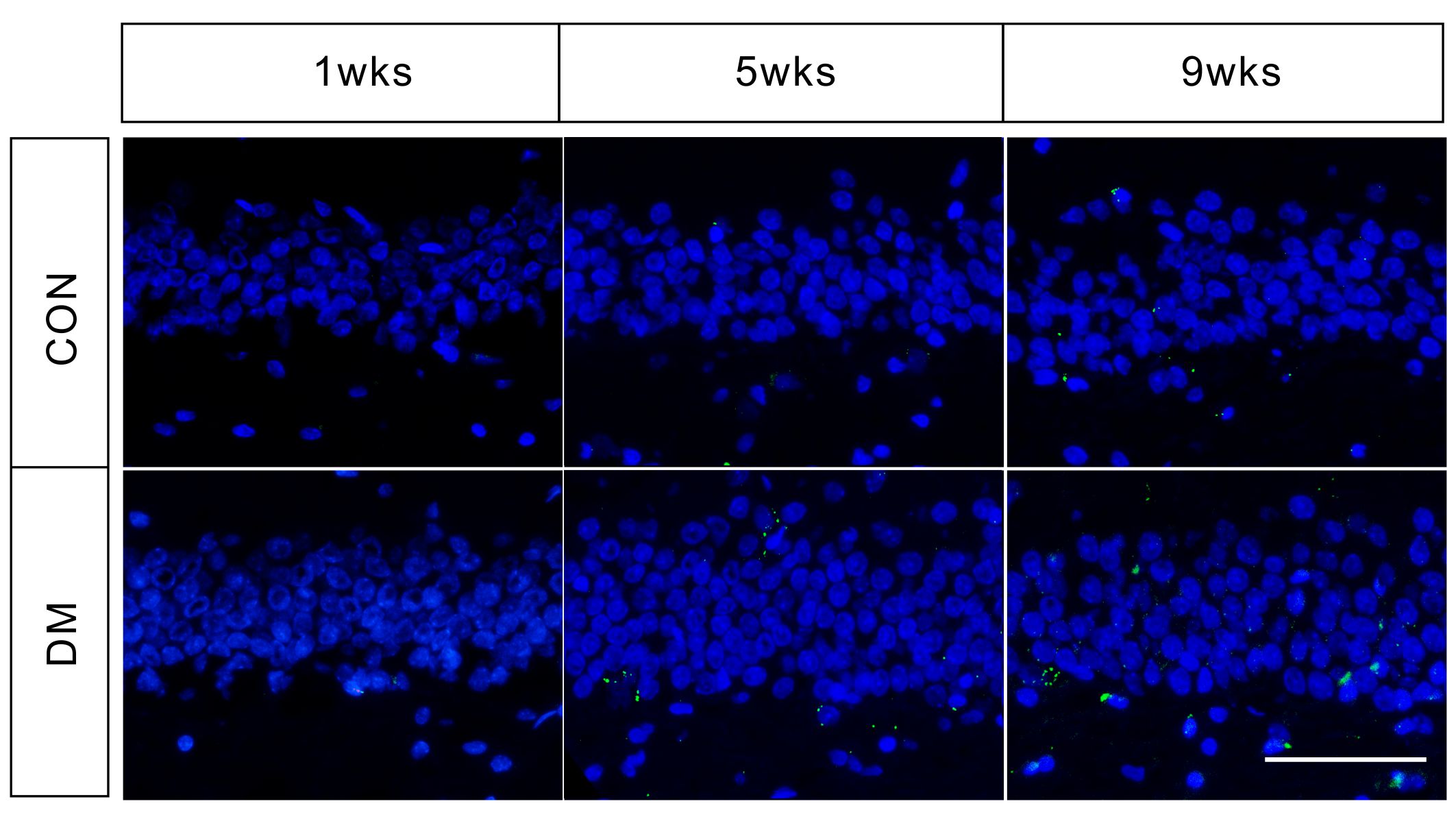


**Supporting Figure 2.** TUNEL staining in the DG regions of hippocampus from STZ-induced diabetic and control rats at different time points. TUNEL-positive cells were stained with green fluorescence, implied with white arrow. Cell nuclei were counterstained blue with DAPI. Scale bar = 50 μm.


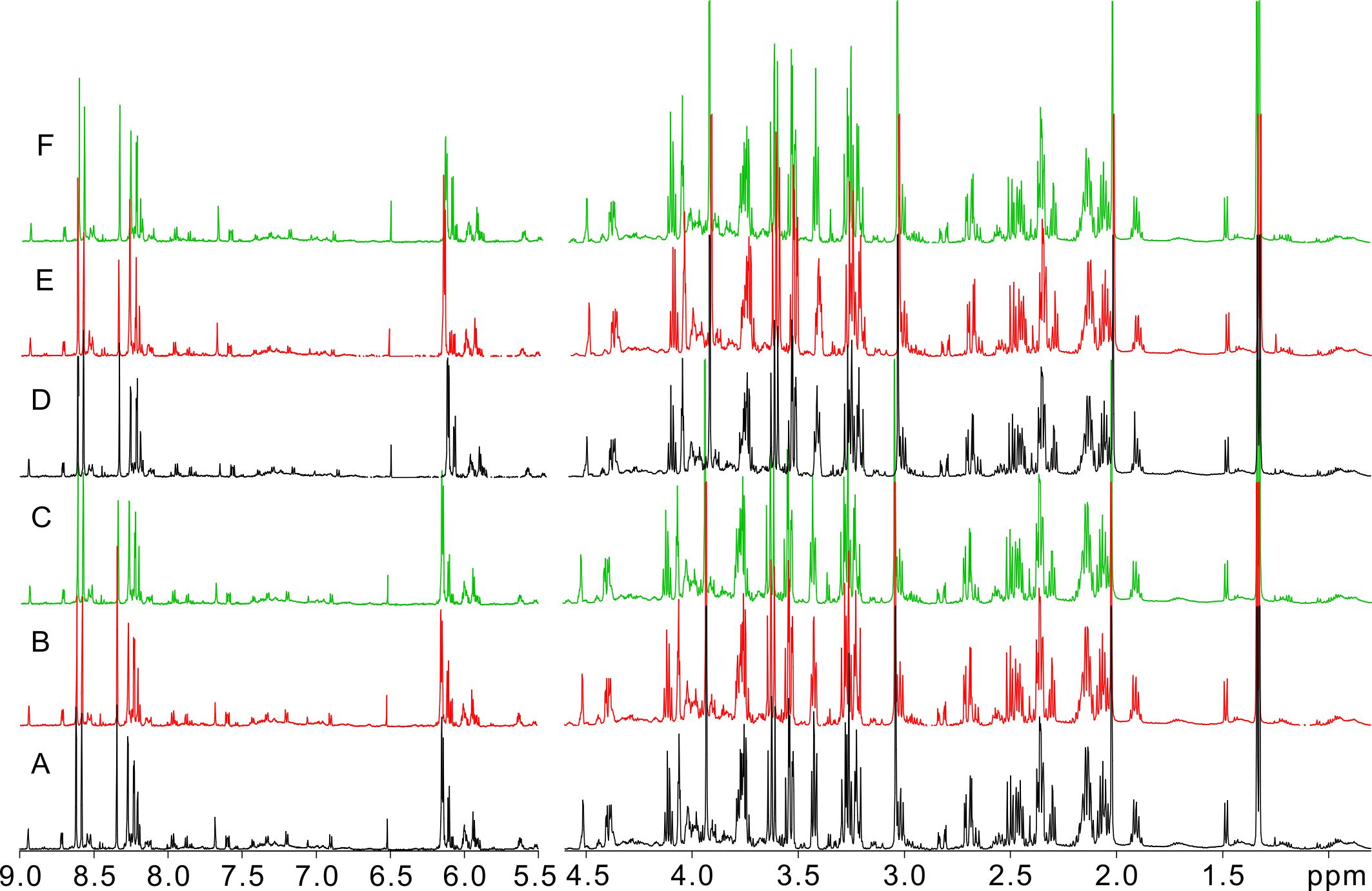


**Supporting Figure 3.** Representative ^1^H-NMR spectra of hippocampus samples obtained from one control 1-week (A), 5-week (B) , 9-week (C) rat, one diabetic 1-week (D), 5-week (E) , 9-week (F) rat, respectively.


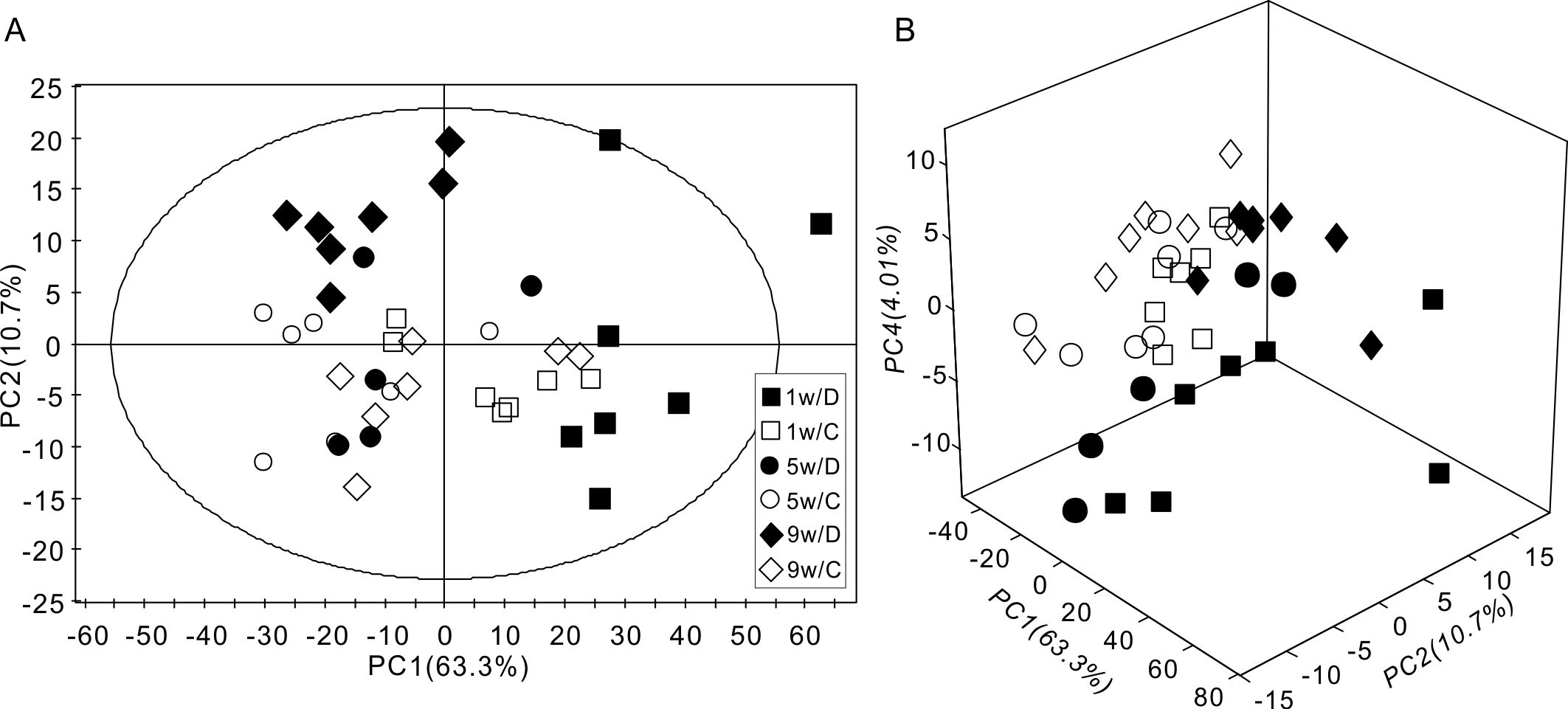


**Supporting Figure 4**. 2D-PCA scores plot (PC1 versus PC2, A) and 3D-PCA scores plot (PC1 versus PC2 versus PC4, B) of ^1^H-NMR data deriving from the hippocampus tissue in three time point of the diabetic group and control group.


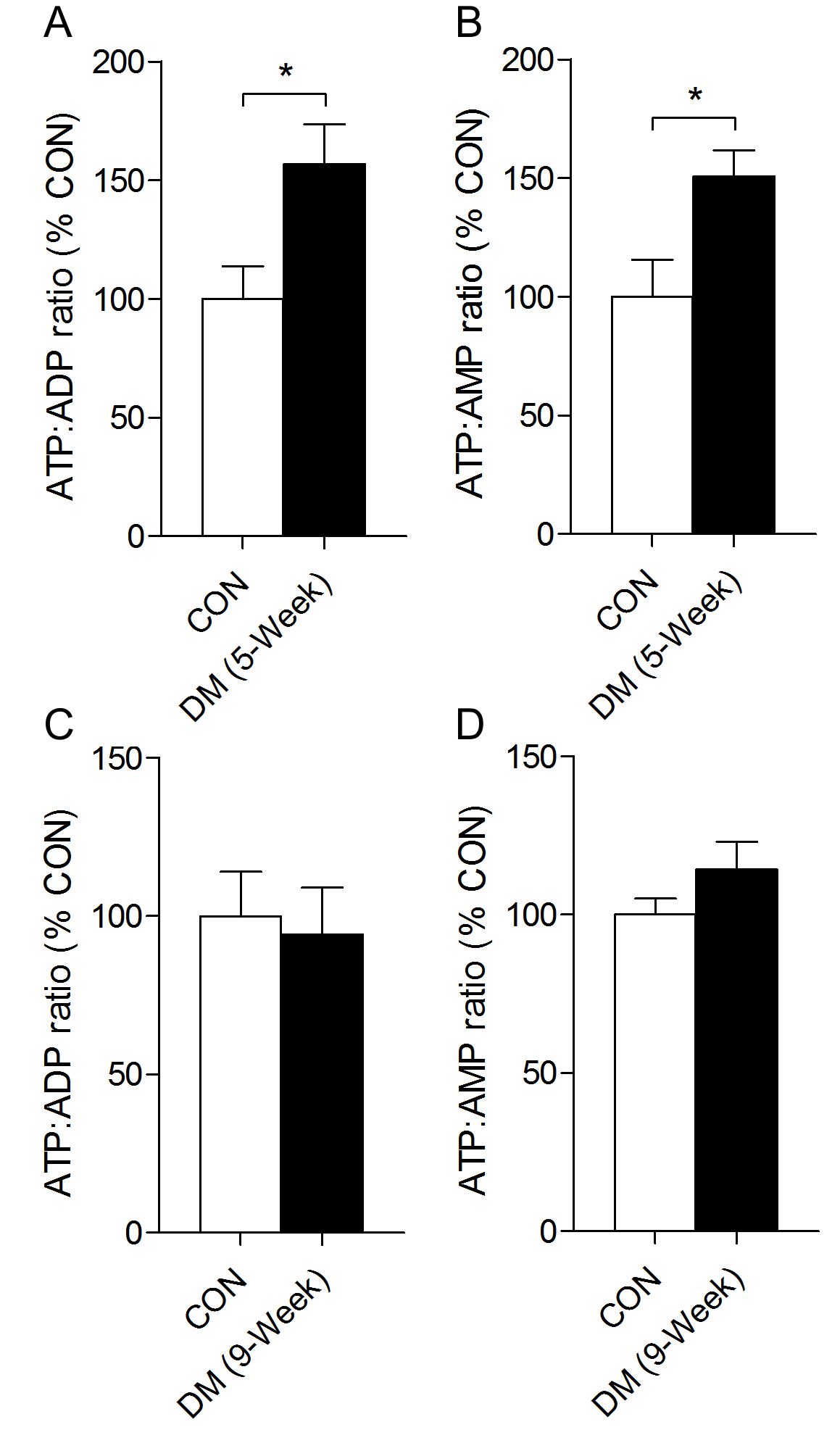


**Supporting Figure 5**. Ratios of ATP/ADP and ATP/AMP in hippocampal tissues from diabetic 5- and 9-weeks rats compared to controls (100% of controls).


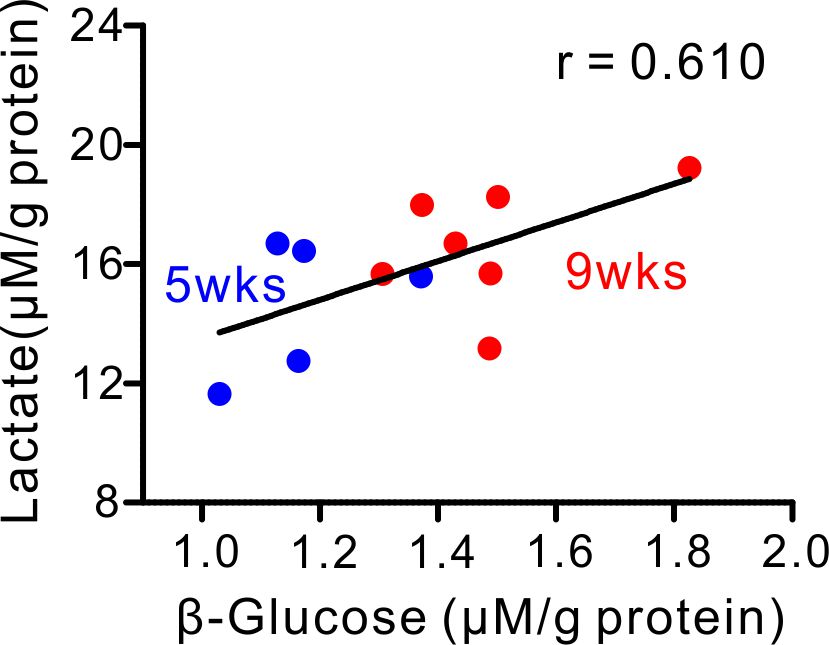


**Supporting Figure 6.** Linear regression analysis was performed on hippocampal β-glucose and lactate in diabetic rats following 5 (blue) and 9 weeks (red) after STZ injection (r = 0.610, p = 0.035).


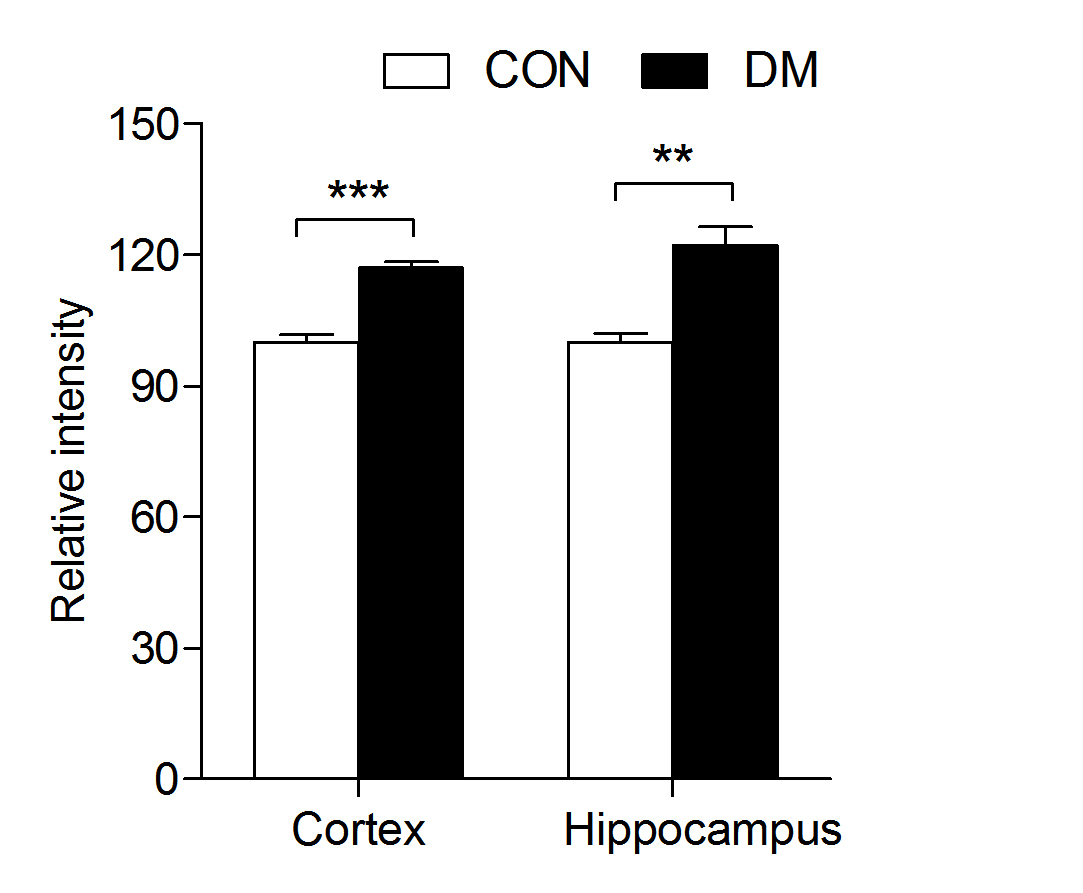


**Supporting Figure 7.** Quantitative analysis of HCA1 expressions of the hippocampus and cortex tissues at 9-week-stages of diabetic and control rats. The relative fluorescence intensity data were acquired by image processing software and counted randomly from 9 fields of each sample slice.


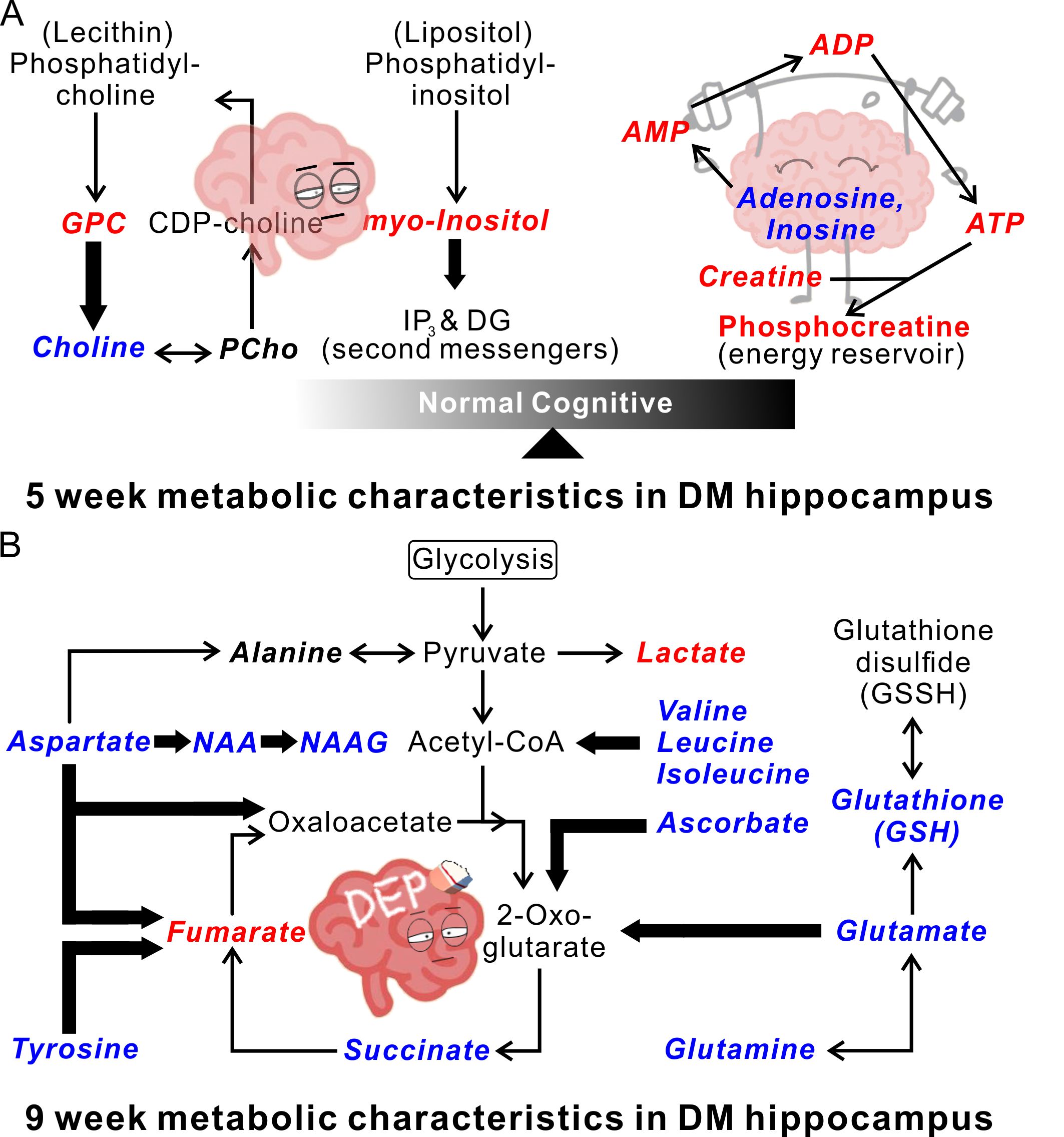


**Supporting Figure 8**. Summary of time-depended metabolic alterations in diabetic rats, compared to age-matched controls. In diabetic 5-week rats, elevated levels of energy reservoir, i.e. ADP, ATP, AMP, phosphocreatine, as well as decreased levels choline were found in the hippocampus (A). In diabetic 9-week rats, reduced contents of most of amino acids and organic acids, conversely, only lactate and fumarate were found increased in the hippocampus (B). Metabolites detected in this study was shown in bond italic font. The red or blue fonts represent increase or decrease in levels, respectively, compared to age-matched controls. Metabolites in black with italic style mean no changes between the two groups.
